# Persistent Down-Beating Torsional Positional Nystagmus: Posterior Semicircular Canal Light Cupula?

**DOI:** 10.1155/2016/1249325

**Published:** 2016-09-07

**Authors:** Akihide Ichimura, Koji Otsuka

**Affiliations:** ^1^Department of Otorhinolaryngology, Tokyo Medical University, 6-7-1 Nishishinjuku, Shinjuku-ku, Tokyo 160-0023, Japan; ^2^Ichimura ENT Clinic, 2-11-10 Nishiwaseda, Shinjuku-ku, Tokyo 169-0051, Japan

## Abstract

A 16-year-old boy with rotatory positional vertigo and nausea, particularly when lying down, visited our clinic. Initially, we observed vertical/torsional (downward/leftward) nystagmus in the supine position, and it did not diminish. In the sitting position, nystagmus was not provoked. Neurological examinations were normal. We speculated that persistent torsional down-beating nystagmus was caused by the light cupula of the posterior semicircular canal. This case provides novel insights into the light cupula pathophysiology.

## 1. Introduction

In the head-hanging position, positional down-beating nystagmus (p-DBN) generally occurs in patients with a cerebellar nodulus lesion [1]. Several authors have recently reported that nystagmus of benign paroxysmal positional vertigo of the anterior semicircular canal (A-BPPV) is observed as a down-beating component with or without a torsional component in the head-hanging position on Dix-Hallpike test [2–4]. In A-BPPV, nystagmus is typically observed as transient positional nystagmus with latency and habituation [2–4]. We report a case of a patient with persistent torsional DBN in the head-hanging position, without central nervous system findings, on the Dix-Hallpike test. We speculated that persistent torsional DBN was not caused by A-BPPV but by the light cupula of the posterior semicircular canal. The condition of this light cupula, characterized by a lower specific gravity than the endolymph, reportedly explains direction-changing characteristics of the first phase of positional alcohol-induced nystagmus with changes in head positions [5, 6]. Several authors have reported that persistent geotropic direction-changing positional nystagmus with the neutral position when turning the head to either side in the supine position occurred because of the light cupula of the horizontal semicircular canal [7–9]. We found that the condition of the light cupula may occur not only in the horizontal but also in the posterior semicircular canal.

## 2. Case Report

**Figure 1:**
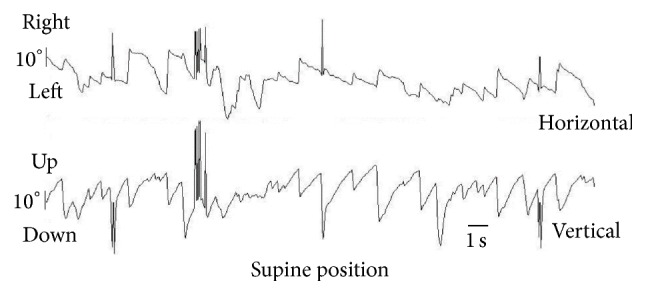
Video-oculographic recording of torsional down-beating nystagmus in the supine position in the head roll test. The vertical component is observed to be down-beating (slow phase velocity 16.5°/s, 84 beats/min) on the vertical recording. The torsional component is observed as a horizontal component beating toward the left on the horizontal recording. Video-oculography was performed using the public domain software ImageJ and a Windows computer [10].

A 16-year-old boy with rotatory positional vertigo and nausea particularly when lying down and at the time of rising visited our clinic on the next day of onset. He denied any hearing loss, tinnitus, headache, or facial neurological symptoms. Past medical, surgical, and family history and head trauma were unremarkable. There was no dysdiadochokinesis, dysmetria, or tremors. Gait was not ataxic, and there was no spontaneous or gaze-evoked nystagmus. Pure tone audiogram, neurological, and eye movement examinations, including the eye-tracking test, saccades, and drum optokinetic nystagmus test, were normal. Brain magnetic resonance imaging (MRI) and magnetic resonance angiography (MRA) findings were normal. The positional and positioning nystagmus test, including the supine head roll and bilateral Dix-Hallpike tests, was recorded using an infrared charge-coupled device camera. The supine head roll test revealed DBN with the torsional component toward the left without latency in straight and right supine positions (Figure 1). The duration of the positional nystagmus was observed for >90 s. The Dix-Hallpike test of the right head-hanging position provoked DBN with the torsional component toward the left for >30 s. In the supine position with the head turning to the left, both left head-hanging and sitting positions on the Dix-Hallpike test did not provoke nystagmus. After 5 days, nystagmus and vertigo disappeared without medical or physical treatment.

The authors have obtained written informed consent from participant's guardian.

## 3. Discussion

We speculated that persistent torsional DBN occurred because of the light cupula of the right posterior semicircular canal in the patient.

Persistent geotropic direction-changing positional nystagmus with the neutral position when turning the head to either side in the supine position reportedly occurred because of the light cupula of the horizontal semicircular canal [7–9]. In the neutral position, the deflectable cupula is almost positioned perpendicular to the gravitational direction without any deflection; therefore, nystagmus is not induced in the neutral position [7–9]. The pathophysiology of the light cupula remains unclear, and there are no reports regarding the light cupula of the posterior semicircular canal. However, Ichijo [8] conjectured that the cupula is deflected by the buoyancy of attached light debris, which has lower specific gravity than the endolymph, such as monocytes and lymphocytes. Furthermore, an increase in the specific gravity of the endolymph may lead to the light cupula [7, 9]. This indicates that the light cupula of the posterior semicircular canal is extremely rare because light debris is more difficult to sink into the ampulla of the posterior semicircular canal in the lower position than into the ampulla of other semicircular canals in sitting and supine positions, if the light cupula is due to the buoyancy of attached light debris.

**Figure 2:**
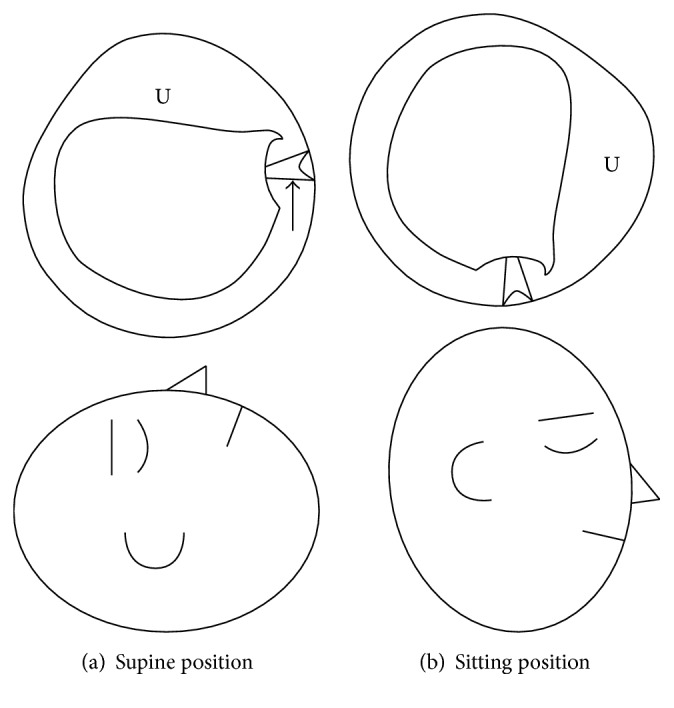
A light cupula of the right posterior semicircular canal. Arrows indicate the deflection of the cupula. U: utricle.

In the light cupula of the posterior semicircular canal, persistent DBN with the torsional component toward the unaffected ear was observed in the affected ear-down position in the supine head roll test, because this position causes ampullopetal deflection of the cupula according to Ewald's third law (Figure 2(a)). Furthermore, nystagmus was not observed in sitting and unaffected ear-down supine positions, because the direction of the deflectable cupula was almost positioned perpendicular to the gravitational direction without any deflection (Figure 2(b)). These positions without nystagmus are regarded as neutral positions in the light cupula of the posterior semicircular canal. Therefore, we speculated that positional nystagmus in our patient was due to the light cupula of the posterior semicircular canal and diagnosed that the right ear was affected.

Several studies have reported that nystagmus of A-BPPV had down-beating component with or without a torsional component in the head-hanging position on the Dix-Hallpike test [2–4]. A-BPPV is generally accompanied by positional nystagmus with typical characteristics of latency, crescendo, and transience [2–4]. Moreover, persistent torsional DBN in the head-hanging position on the Dix-Hallpike test reportedly occurred by the anterior canal cupulolithiasis, a rare variant of A-BPPV [11–13]. However, the pathophysiology of nystagmus also involves the anterior canal cupulolithiasis, which raises several concerns. First, in patients with persistent torsional DBN, reversal of nystagmus was not observed while shifting from the head-hanging to sitting position. Second, nystagmus was not observed as persistent positional nystagmus in the sitting position. In anterior canal cupulolithiasis, the debris should rest on the cupula in the sitting position because the direction of the deflectable cupula is almost positioned to the gravitational direction in the sitting position; this theoretically induces persistent upbeating torsional nystagmus because of ampullopetal deflection of the cupula according to Ewald's third law. There are no reports regarding anterior canal cupulolithiasis with persistent upbeating torsional nystagmus in the sitting position, indicating that cupulolithiasis is not the cause of nystagmus.

Vannucchi et al. reported that torsional DBN in the head-hanging position in the Dix-Hallpike test also occurred because of a rare variant canalolithiasis of the posterior semicircular canal [14]. Their hypothesis involved the debris being in the highest part of the posterior canal in the sitting position and dislodging toward the ampulla in the long arm in the bilateral Dix-Hallpike positions [14]. However, it is difficult in this theory to explain persistent torsional DBN because of canalolithiasis, and nystagmus is not observed while shifting from the head-hanging to the sitting position.

P-DBN in the head-hanging position, with or without slight positional vertigo, is indicative of a cerebellar nodulus lesion and may be caused by multiple sclerosis, ischemia, intoxication, craniocervical malformation, or cerebellar degeneration [1]. However, neurological examination and brain MRI/MRA findings were normal in the patient. The patient was 16 years old and did not experience any gaze-evoked nystagmus. Nystagmus and rotatory vertigo disappeared after 5 days. Thus, we speculated that central nervous system disorders do not cause nystagmus.

In conclusion, we speculated that persistent torsional DBN in our patient was due to the light cupula of the posterior semicircular canal. We determined that the condition of the light cupula probably occurred not only in the horizontal but also in the posterior semicircular canal. These findings prove useful for elucidating the light cupula pathophysiology.

## Supplementary Material

Video1. Positional nystagmus in the supine position: The supine head roll test revealed DBN with the torsional component toward the left without latency in straight supine position. The duration of the positional nystagmus was observed for > 90 s.
Video2. Positioning nystagmus: The Dix–Hallpike test of the right head-hanging position provoked DBN with the torsional component toward the left for > 30 s. Both left head-hanging and sitting positions on the Dix–Hallpike test did not provoke nystagmus.
